# High Polyunsaturated Fatty Acid Intake Attenuates the Genetic Risk of Higher Waist Circumference in a Sri Lankan Adult Population

**DOI:** 10.3390/nu17172866

**Published:** 2025-09-04

**Authors:** Padmini Sekar, Julie A. Lovegrove, Shelini Surendran, Karani Santhanakrishnan Vimaleswaran

**Affiliations:** 1 Hugh Sinclair Unit of Human Nutrition, Department of Food and Nutritional Sciences, Institute for Cardiovascular and Metabolic Research (ICMR), University of Reading, Reading RG6 6DZ, UK; p.sekar@pgr.reading.ac.uk (P.S.); j.a.lovegrove@reading.ac.uk (J.A.L.); 2 The Institute for Food, Nutrition, and Health (IFNH), University of Reading, Reading RG6 6AH, UK; 3 Faculty of Health and Medical Sciences, University of Surrey, Guildford GU2 7XH, UK; s.surendran@surrey.ac.uk

**Keywords:** Sri Lanka, genetic risk score, single nucleotide polymorphism, gene-diet interaction, metabolic diseases

## Abstract

Background: Metabolic diseases, like type 2 diabetes mellitus and obesity, show a growing public health concern in Sri Lanka. Genetic predisposition and diet contribute to metabolic disease risk, but there are limited investigations into the impact of gene–diet interactions on metabolic disease risk in the Sri Lankan population. In this study, we examined whether a metabolic genetic risk score (GRS), constructed from 10 single nucleotide polymorphisms (SNPs), interacts with dietary factors to influence metabolic health indicators in Sri Lankan adults. Methods: This cross-sectional study included 105 generally healthy adults aged 25–50 years from the GOOD (Genetics of Obesity and Diabetes) study. Anthropometric, biochemical, and dietary data using food frequency questionnaires were collected using validated methods. Genotyping was performed using the KASP^®^ assay. The unweighted GRS was calculated by summing risk alleles across 10 SNPs in the *TCF7L2*, *CAPN10*, *FTO KCNJ11*, and *MC4R* genes. Gene–diet interaction analysis was conducted using regression models adjusted for confounders. Results: A statistically significant interaction was identified between the 10-SNP metabolic GRS and polyunsaturated fatty acid (PUFA) intake on waist circumference (P_(interaction)_ = 0.00009). Participants with a high GRS (≥6 risk alleles) and higher PUFA intake (≥3.1 g/day) exhibited significantly lower waist circumference (*p* = 0.047). Conclusions: This study provides novel insights to understand gene–diet interactions affecting metabolic traits in Sri Lankans. The findings suggest that higher PUFA intake may mitigate genetic susceptibility to central obesity, highlighting the importance of personalized dietary recommendations for metabolic disease prevention. Further studies in larger cohorts are warranted to confirm this finding.

## 1. Introduction

Metabolic diseases, including obesity and type 2 diabetes, are characterised by metabolic dysfunction, leading to conditions such as insulin resistance, hyperglycaemia, and dyslipidaemia [1]. These disorders result from a complex interplay of genetic, environmental, and lifestyle factors, including sedentary behaviour and poor diet [2]. In South Asia, a region predominantly comprising low- and middle-income countries, the prevalence of metabolic diseases has risen alarmingly [2]. The global burden of metabolic diseases, including type 2 diabetes mellitus and hypertension, has grown significantly from 2000 to 2019, with the highest mortality rates observed in low to low-middle socio-demographic index countries [3]. Shifts in urban development, economic growth, and lifestyle have led to rising cases of obesity and type 2 diabetes mellitus, creating major public health issues and pressuring health infrastructure [2].

In Sri Lanka, a country with a population of 22 million, obesity and type 2 diabetes mellitus prevalence has markedly increased [3,4]. A nationally representative study reported that the prevalence of overweight and obesity among Sri Lankan adults was 25.2% and 9.2%, respectively, based on Asian-specific body mass index (BMI) cut-off values [5]. Additionally, a cross-sectional descriptive study conducted in the Central Province of Sri Lanka found that 32.3% of men were overweight, and 13.2% were obese, emphasising the considerable impact of obesity in this setting [6]. A systematic review and meta-analysis conducted in Sri Lanka reported a pooled prevalence of type 2 diabetes mellitus of 11.8% in the 2000s, increasing to 17.3% during 2011–2021, highlighting a growing trend over the last three decades [4]. The same study also showed that the pooled prevalence of type 2 diabetes mellitus in Sri Lanka increased from 5.6% in the 1990s to 17.3% in the 2011–2021 period, underscoring the urgency for effective interventions [4].

The escalation of overweight and obesity in Sri Lanka can be attributed to factors such as sedentary lifestyles, unhealthy diets, and genetic predisposition. A study assessing dietary patterns among Sri Lankan adults found that individuals with higher dietary diversity scores tended to have increased obesity measures. This may be due to greater consumption of energy-dense foods, resulting in higher overall caloric intake and body weight [6]. Furthermore, research has identified genetic markers that are significantly associated with increased susceptibility to obesity in studies conducted among Sri Lankan populations. For example, variants of the fat mass and obesity-associated gene (*FTO*) and melanocortin 4 receptor (*MC4R*) genes have been shown to be associated with higher BMI and obesity measures, with urban living amplifying the effect of the *FTO* polymorphism [7]. Additionally, metabolic disorders are also prevalent among Sri Lankan children, even those not classified as obese by anthropometric measures, emphasizing the importance of early detection and intervention [8]. Previous studies have examined gene–diet interactions on vitamin B12 status in the Sri Lankan population [9,10]. Nonetheless, few studies have directly explored how metabolic GRS interacts with dietary intake in influencing obesity and type 2 diabetes mellitus-related traits. Hence, we have constructed a unique 10-single-nucleotide polymorphism (SNP) metabolic genetic risk score (GRS) to investigate GRS–diet interactions on metabolic-disease-related traits in the Sri Lankan population.

## 2. Materials and Methods

### 2.1. Study Participants

The GOOD (Genetics of Obesity and Diabetes) study is a cross-sectional study conducted in Colombo, Sri Lanka, from April to August 2017 [9]. Healthy individuals aged 25 to 50 years were enrolled in this study. Approval for this study was granted by the Ethics Committee of the University of Colombo (EC-17-107) and the University of Reading Research Ethics Committee (17/25) on the 15th of February 2017. Written informed consent was obtained from each participant before taking part in the study. Participants were excluded if they had a prior diagnosis of type 2 diabetes mellitus, cardiovascular disease, or hypertension. Additional exclusion criteria included a BMI exceeding 40 kg/m^2^ or a classification of morbid obesity by a physician, being related biologically to other study participants, currently presenting with communicable disease, women who are lactating or pregnant, using dietary or medical supplements, or taking medications that influence lipid metabolism or blood pressure.

### 2.2. Anthropometric Measures

An electronic scale (Seca 815, Seca GmbH, Hamburg, Germany) was used to measure body weight to the nearest 100 g, and height was assessed using a stadiometer (Seca 217, Seca GmbH, Germany) accurate to the nearest millimetre. BMI was calculated as body weight (in kg) divided by the square of height (in m). Body fat percentage estimation was performed using a bioelectrical impedance handheld analyser (Omron Body Fat Monitor BF306, Omron, Milton Keynes, UK). A flexible measuring tape (Lufkin W606PM^®^, Parsippany, NJ, USA) was used to obtain waist and hip circumference measurements.

### 2.3. Biochemical and Clinical Measures

Following a 12 h overnight fast, a trained phlebotomist collected 10 mL of blood for analysis. Fasting serum insulin was measured using the chemiluminescent microparticle immunoassay method on an Architect i1000 analyser (Abbott Laboratories, Abbott Park, IL, USA). Glycated haemoglobin (HbA1c) levels were quantified through high-performance liquid chromatography (HPLC) using the BioRad D10 HPLC analyser (BioRad, Hercules, CA, USA). Plasma fasting glucose levels were assessed using the glucose hexokinase method using a Beckman Coulter AU5800 analyser (Beckman Coulter^®^, Brea, Orange County, CA, USA).

### 2.4. Dietary Assessment

An interviewer-administered, validated food frequency questionnaire (FFQ) listing 85 food items was used to evaluate participants’ dietary intake [11]. Participants reported their typical consumption patterns by specifying how often they consumed each item (daily, weekly, monthly, or never) along with an estimation of portion sizes. This dietary data was analysed using the NutriSurvey 2007 database (EBISpro, Willstatt, Ortenau, Germany) to estimate total energy, macronutrient, and micronutrient intakes [12].

### 2.5. SNP Selection and Genotyping

For this study, 10 metabolic-disease-related SNPs (association with obesity and/or type 2 diabetes mellitus), Calpain-10 (*CAPN10*) rs2975760, rs5030952 [13] and rs3792267 [14], Potassium inwardly rectifying channel, subfamily J, member 11 protein (*KCNJ11*) rs5219 [15], Transcription factor 7 like 2 (*TCF7L2*) rs12255372 and rs7903146 [16,17,18], Fat mass and obesity-associated (*FTO*) rs9939609 and rs8050136 [19,20,21], near Melanocortin-4 receptor (*MC4R*) rs17782313 [22], and Melanocortin-4 receptor (*MC4R*) rs2229616 [23], were screened and selected based on previously published candidate gene association and genome-wide association studies (GWAS) for metabolic-disease-related traits.

Blood samples intended for DNA analysis were transported to the UK on dry ice. Genomic DNA was extracted from 5 mL of whole blood collected from each participant. Genotyping was carried out at LGC Genomics using the Kompetitive Allele-Specific PCR (KASP^®^) platform (https://www.biosearchtech.com/products/oligos-probes-and-primers/kasp-genotyping-assays/, accessed on 9 January 2025).

The *p*-values of Hardy–Weinberg equilibrium (HWE) was calculated for all the 10 SNPs chosen for the study. The CAPN10 SNP rs3792267 showed deviation from HWE (*p* = 0.05); however, it was retained in the analysis. This deviation is unlikely to be due to genotyping error, as the KASP™ platform used in this study has a validated accuracy rate exceeding 99.8%, and all genotyping was independently reviewed by project managers at LGC Genomics. The observed deviation may instead reflect true biological phenomena such as population stratification or selection. Additionally, rs3792267 has well-established associations with insulin resistance and type 2 diabetes in South Asian and other populations [14,24], making it biologically relevant to the construction of the metabolic GRS. Excluding this variant would weaken the comprehensiveness of the risk score [14,24,25]. The KASP™ genotyping technology used in this study has been independently verified to exhibit an accuracy rate exceeding 99.8%. Validation was carried out at LGC Genomics, where two project managers independently reviewed the genotyping results to ensure data accuracy. This thorough assessment eliminated genotyping artifacts as a potential cause for deviations from Hardy–Weinberg equilibrium (HWE).

### 2.6. Statistical Analysis

Data were statistically analysed using SPSS version 27 (SPSS Inc., Chicago, IL, USA). Allele frequencies were determined through gene counting. Normality of the data distribution was assessed using the Shapiro–Wilk test, and variables that deviated from normal distribution were log-transformed before further analysis [age, BMI, waist circumference (WC), hip circumference, systolic blood pressure, diastolic blood pressure, HDL, VLDL, glucose, insulin, HbA1c, total energy intake, protein intake, carbohydrate intake, fat intake, fibre intake, polyunsaturated fatty acid (PUFA) intake]. An unweighted GRS was calculated for each participant by summing the number of risk alleles across all SNPs, applying an additive genetic model. The 10-SNP metabolic GRS was derived from SNPs in the *CAPN10*, *KCNJ11*, *TCF7L2*, *FTO*, and *MC4R* genes, which have been linked to metabolic disorders. An unweighted GRS was calculated for each participant by summing the number of risk alleles across all SNPs, applying an additive genetic model. This method was selected due to the absence of established effect sizes for these SNPs in South Asian populations. Each SNP was assigned a value of 0, 1, or 2, representing the number of risk alleles present. The total GRS for each participant was calculated by summing the risk alleles across all SNPs. The median number of risk alleles per individual was 6.00. Based on this, participants were classified into two groups: a low genetic risk group (GRS < 6 risk alleles, *n* = 50) and a high genetic risk group (GRS ≥ 6 risk alleles, *n* = 55). Participants were also grouped based on central obesity status (centrally obese: WC ≥ 94 cm for men; WC ≥ 80 cm for women). Independent t-tests were applied to compare continuous variables across genetic risk categories and central obesity status (WC ≥ 94 cm for men; WC ≥ 80 cm for women). Association between GRS and anthropometric, biochemical, and dietary parameters was performed using linear regression analysis. Categorical variables were analysed using the chi-square test to compare distributions between the two groups.

To evaluate gene–diet interactions, interaction terms (GRS × dietary intake) were incorporated into regression models. The interaction models were adjusted for age, sex, BMI, total energy intake, smoking and alcohol intake, where applicable. Since there were no prior studies examining this 10-SNP metabolic GRS or reporting effect sizes in South Asian populations, a power calculation could not be conducted. In accordance with recent recommendations, post hoc power analysis was not conducted, as it is considered statistically uninformative and potentially misleading [26]. Instead, we report the observed R^2^ value from the interaction regression model (R^2^ = 0.061), which reflects the variance in waist circumference explained by the genetic risk score, PUFA intake, and their interaction term. This approach provides a more accurate reflection of model performance in the context of gene–diet interaction analysis.

## 3. Results

### 3.1. Association of Anthropometric, Biochemical and Dietary Characteristics with Central Obesity

Participants were categorised based on central obesity status (centrally obese: WC ≥ 94 cm for men; WC ≥ 80 cm for women). Statistically significant associations were observed between the two groups in metabolic-disease-related traits such as BMI (*p* ≤ 0.001), waist circumference (*p* = 0.039), fat mass (*p* ≤ 0.001), systolic blood pressure (*p* = 0.019), and insulin levels (*p* ≤ 0.001) (Table 1).

### 3.2. Association of GRS with Anthropometric, Biochemical and Dietary Characteristics

A total of 105 individuals were included in this study. Table 2 presents comparisons of anthropometric, biochemical, and dietary characteristics between low and high GRS groups. Supplementary Table S1 shows the allele frequency and HWE for the SNPs in the study population.

### 3.3. Interaction Between 10 SNP Metabolic GRS and Dietary Factors on Anthropometric and Biochemical Parameters

**Figure 1:**
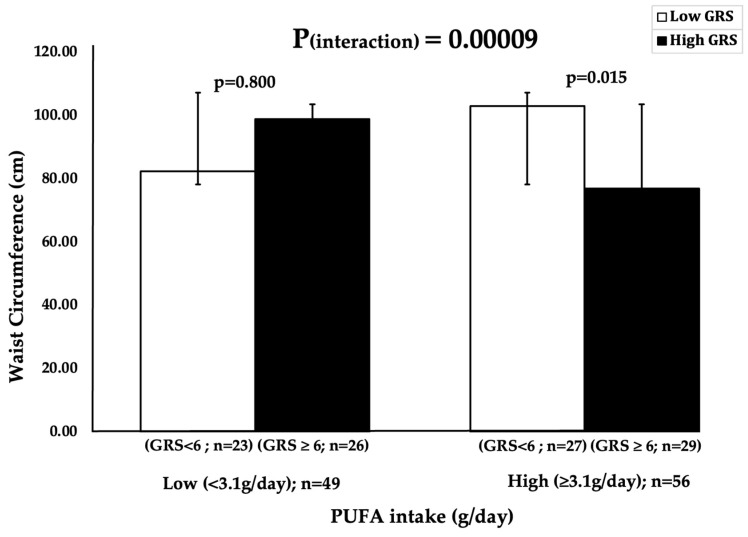
Binary analysis for the interaction between 10-SNP metabolic GRS and PUFA (%) intake on waist circumference (cm). Participants were stratified by PUFA intake using a median cutoff of 3.1 g/day (log-transformed value ≈ 0.49). Participants were stratified into four groups: Low GRS/Low PUFA (*n* = 27); Low GRS/High PUFA (*n* = 23); High GRS/Low PUFA (*n* = 21); High GRS/High PUFA (*n* = 34). This threshold reflects typical intake levels among Sri Lankan adults in this study and was chosen for internal stratification. Low GRS (<6 risk alleles); high GRS: (≥6 risk alleles).

Four significant interactions were observed between the 10-SNP metabolic GRS and dietary factors on metabolic disease-related traits (Table 3). Further stratification of participants was performed based on dietary intake of study parameters into high and low intake; statistically significant association (*p* = 0.015) was only observed for the interaction between the GRS and PUFA intake on waist circumference P_interaction_ ≤ 0.001 (0.00009) (Figure 1). Binary analysis indicated that individuals with high PUFA intake (≥3.1 gm/day) and having high GRS (≥6 risk alleles) had a statistically significant lower waist circumference (*p* = 0.047) compared to the low genetic risk counterparts. When stratified by PUFA intake groups, participants in the high PUFA group exhibited a lower mean waist circumference (81.56 ± 2.79 cm) compared to those in the low PUFA group (85.82 ± 2.06 cm). However, this difference was not statistically significant (*p* = 0.125). Yet, this trend supports the observed gene–diet interaction, wherein individuals with higher genetic risk demonstrated significantly lower waist circumference when consuming higher levels of PUFA.

## 4. Discussion

The study explored the interaction between a 10-SNP metabolic GRS and dietary factors on metabolic-disease-related traits in a Sri Lankan population. A statistically significant interaction was observed between the 10-SNP metabolic GRS and PUFA intake on waist circumference P_interaction_ ≤ 0.001 (0.00009), whereby individuals with higher PUFA intake (≥0.49 g/day) and a high GRS (≥6 risk alleles) exhibited significantly lower waist circumference (*p* = 0.015) than their counterparts with lower genetic risk. To our knowledge, this is the first study in Sri Lanka to examine gene–diet interactions using this 10-SNP metabolic GRS. These results highlight the need to account for genetic susceptibility alongside dietary habits when evaluating central obesity risk. From a public health perspective, this evidence highlights the potential of integrating genetic risk assessment into nutritional guidelines. Encouraging PUFA-rich dietary patterns in genetically predisposed individuals may represent an effective intervention to reduce obesity and metabolic disease prevalence in Sri Lanka. As metabolic diseases continue to rise, incorporating precision nutrition strategies into public health policies may offer more effective, sustainable solutions to combat metabolic diseases, tailored to the genetic and dietary contexts of the local population.

The ten SNPs selected for constructing the metabolic GRS were chosen based on both their established associations with metabolic diseases and their functional roles in relevant biological pathways, as detailed below. Variants in the *CAPN10* gene (rs3792267, rs2975760, and rs5030952) have been associated with impaired insulin secretion and increased type 2 diabetes mellitus risk across multiple ethnic groups, including Asians [14,24]. The *KCNJ11* rs5219 variant has been shown to affect pancreatic β-cell function and implicated in type 2 diabetes mellitus susceptibility [15]. Similarly, *TCF7L2* polymorphisms (rs12255372 and rs7903146) are among the strongest genetic risk factors for type 2 diabetes mellitus, influencing insulin secretion and glucose homeostasis [16,27,28]. In relation to obesity, the *FTO* variants (rs9939609 and rs8050136) [19] and *MC4R* variants (rs17782313 and rs2229616) have been widely recognized for their roles in regulating appetite and energy balance [29]. Importantly, studies conducted in Sri Lankan and broader South Asian populations have demonstrated associations between *FTO* and *MC4R* variants and increased adiposity, highlighting the relevance of these loci in this ethnic context [7]. Given the alarming rise in obesity and type 2 diabetes mellitus in Sri Lanka, largely driven by rapid lifestyle changes, understanding the genetic underpinnings is crucial [3,4]. Therefore, selecting SNPs with established functional significance in metabolic pathways ensures that the GRS reflects the biological mechanisms underlying metabolic disease risk in this population. Moreover, a multi-SNP GRS approach offers a more comprehensive capture of genetic susceptibility compared to analysing single variants [30], thereby providing valuable insights for developing precision nutrition strategies targeted at South Asians.

In this study, we identified a significant interaction between PUFA intake and the 10-SNP metabolic GRS on waist circumference, suggesting that higher PUFA consumption may attenuate the adverse effects of genetic predisposition to central obesity. Notably, none of the individual SNPs included in the GRS showed significant associations with metabolic traits when analysed independently using logistic regression (Supplementary Table S2). This underscores the advantage of employing a cumulative GRS, which can better capture the polygenic architecture of complex traits. The significant interaction observed between the GRS and PUFA intake suggests that the combined genetic burden may have a more pronounced effect on metabolic outcomes than any single variant alone, particularly in the context of dietary modulation. Additionally, when stratified by PUFA intake, participants in the high PUFA group had a lower mean waist circumference (81.56 ± 2.79 cm) compared to the low PUFA group (85.82 ± 2.06 cm). However, this difference was not statistically significant (*p* = 0.125). This indicates that PUFA intake alone is not associated with WC in the overall cohort. However, the observed significant gene–diet interaction between PUFA intake and the metabolic GRS on waist circumference (P_interaction_ < 0.001), suggests that the relationship between PUFA intake and waist circumference is influenced by genetic background.

The World Health Organization (WHO) recommends that PUFA intake should contribute 6–11% of total energy intake [31], which translates to approximately 13–24 g/day for an average 2000-calorie diet. This includes both omega-3 and omega-6 fatty acids, emphasizing their role in metabolic health and disease prevention [32,33]. However, participants had a median PUFA intake of only 3.1 g/day and our study’s “high PUFA” group still falls below global recommendations of required daily PUFA intake. It is recommended that higher PUFA intake in the diet could prove beneficial. Our findings align with previous research indicating the beneficial role of PUFAs in modulating obesity risk through mechanisms such as improved lipid metabolism, enhanced insulin sensitivity, and anti-inflammatory effects [34,35,36]. Collectively, these biological effects suggest that PUFA intake may counteract genetic susceptibility to central obesity, as reflected by reduced waist circumference, by promoting favourable metabolic pathways, thereby supporting the gene–diet interaction observed in our study.

Our findings show that high PUFA intake may attenuate the impact of genetic variants predisposing to central adiposity. Several genes included in the 10-SNP metabolic GRS—notably *FTO*, *MC4R*, *TCF7L2*, *CAPN10*, and *KCNJ11*—influence pathways related to appetite regulation, adipocyte differentiation, and insulin signalling [37,38,39,40]. For instance, *FTO* and *MC4R* variants were associated with hyperphagia and increased fat storage [29,41], while *TCF7L2* variants affect insulin secretion and glucose metabolism [16]. Through activation of PPAR-γ and stimulation of fatty acid oxidation, PUFAs may counteract the obesogenic effects of these genetic variants by redirecting energy metabolism toward reduced fat deposition and improved insulin action [36,42]. Additionally, PUFAs possess anti-inflammatory properties that may mitigate the chronic low-grade inflammation often observed in individuals with a high genetic risk for central obesity [33,34]. Therefore, higher PUFA intake could function as an environmental modifier, buffering the genetic predisposition to central adiposity and explaining the observed lower waist circumference among participants with high GRS and higher PUFA consumption in our study.

Previous research has demonstrated that dietary fat composition interacts with genetic variants to influence insulin sensitivity and lipid profiles [43,44]. In Sri Lanka, dietary patterns are predominantly characterized by high consumption of carbohydrates, mainly in the form of polished white rice, starchy vegetables, and sugary beverages [12]. Sri Lankan cuisine typically includes rice paired with coconut-milk-based curries, lentils, and small portions of meat or fish, resulting in diets that are energy-dense and rich in saturated fats, while being relatively low in polyunsaturated fats [7,12]. Coconut oil, a commonly used fat in Sri Lankan cuisine, is a major contributor to the elevated saturated fat levels in traditional diets. Coconut oil is widely used for cooking, frying, and as an ingredient in curries and confectionery, leading to a diet rich in saturated fatty acids [12]. Approximately 80–90% of the fatty acids in coconut oil are saturated, primarily lauric acid, which has been associated with elevated LDL cholesterol levels, a well-established risk factor for cardiovascular and metabolic diseases [45]. Although some studies have suggested that coconut oil may increase HDL cholesterol and possesses neutral effects on certain cardiometabolic outcomes, the overall high intake of SFA is concerning, particularly in populations with heightened genetic susceptibility to metabolic diseases [46,47]. Moreover, frequent snacking on sweetened foods and limited intake of fruits and vegetables further contribute to an imbalance in macronutrient distribution [5]. This imbalance results in a diet that is high in carbohydrates and saturated fat, but low in PUFAs—a pattern that has been associated with insulin resistance, increased adiposity, and dyslipidaemia, particularly in South Asian populations [3,48]. High carbohydrate intake, common in South Asian diets, has been linked to increased adiposity, insulin resistance, and dyslipidaemia [3,48]. Conversely, PUFAs have demonstrated protective effects against these metabolic disturbances [49].

Given this dietary context in Sri Lanka [12], our study’s finding that individuals with high genetic risk for metabolic disease had significantly lower waist circumference when consuming higher levels of PUFA is especially relevant. The median PUFA intake observed in our cohort (~3.1 g/day) is substantially lower than WHO’s recommended PUFA intake of 13–24 g/day for a standard 2000 kcal diet [31], reinforcing the need for targeted nutritional interventions. Encouraging the inclusion of locally available PUFA-rich foods may help increase intake levels. In the Sri Lankan context, such sources include small oily fish, like sardines and mackerel, flaxseeds, PUFA-enriched cooking oils (such as soybean or sunflower oil), and eggs fortified with omega-3 fatty acids. Incorporating these foods into daily diets may offer a culturally appropriate means of mitigating genetic risk for central obesity. Given the genetically heightened susceptibility to metabolic disorders in Sri Lankans [7,30] population-wide shifts toward PUFA-rich dietary patterns may not only improve metabolic outcomes but also help to counteract gene-driven obesity risk. These findings support the integration of nutrigenetic insights into public health policies and dietary guidelines for Sri Lanka, enabling the development of culturally relevant and genetically informed interventions to address the burden of central obesity and related non-communicable diseases.

One of the strengths of this study is the use of a GRS approach, that captures the cumulative effect of multiple SNPs rather than focusing on individual genetic variants. This method provides a more comprehensive assessment of genetic predisposition to metabolic diseases. Additionally, this is the first study using this 10-SNP metabolic GRS to examine gene–diet interactions in a Sri Lankan cohort, contributing valuable insights to the field of nutrigenetics in South Asia. However, the limitations should also be acknowledged. First, the sample size for this study was relatively small, which may limit the generalizability of the findings and reduce statistical power to detect additional significant interactions. Larger studies are needed to validate these results in broader Sri Lankan and South Asian populations. Although the relatively modest sample size in our study is a limitation, the post hoc sensitivity analysis conducted using G*Power 3.1 showed that our study was sufficiently powered (77%) to detect small-to-moderate interaction effects. The analysis suggests that the sample size of 105 participants was adequate to observe meaningful gene–diet interaction effects, even within the context of a limited cohort. A key limitation of our study is the modest sample size (*n* = 105), which may reduce the statistical power to detect subtle interaction effects. However, rather than rely on post hoc power calculations, we emphasize the significance of the observed gene–diet interaction and its effect size (R^2^ = 0.061), which supports the biological plausibility of the findings [26]. While larger sample sizes would enhance generalizability and precision, studies with sample sizes in this range can still yield meaningful insights, particularly in exploratory gene–diet interaction studies involving well-defined cohorts. Secondly, although the FFQ used was validated for this population, it is inherently prone to recall bias and misreporting. Consequently, absolute estimates of dietary intake—especially of micronutrients or specific fatty acids like PUFA—may be imprecise. Future studies should consider incorporating objective biomarkers of dietary intake, such as plasma fatty acid levels, to enhance the validity of gene–diet interaction analysis. Another limitation is the use of an unweighted GRS, which does not account for the relative contribution of each SNP. However, given the lack of effect size data for South Asian populations, the unweighted approach avoids bias introduced by applying effect sizes derived from unrelated populations. Finally, the cross-sectional nature of the study precludes causal inferences, emphasizing the need for longitudinal studies to confirm the observed associations.

The findings of this study underscore the potential of personalized nutrition in mitigating metabolic disease risk in genetically susceptible individuals. Given the high burden of metabolic diseases in Sri Lanka, integrating genetic screening with dietary interventions could offer more effective strategies for disease prevention and management. Future studies should explore gene–diet interactions in larger cohorts, incorporate additional dietary components, and examine the long-term effects of dietary modifications on metabolic health.

## 5. Conclusions

This study is the first to report a significant interaction between this 10-SNP metabolic GRS and PUFA intake on waist circumference among a Sri Lankan population. Our findings reveal that individuals with a higher genetic risk of metabolic disease may benefit from higher dietary PUFA intake, as it was linked with a reduction in waist circumference, a core measure of central obesity. These results emphasize the potential of targeted dietary strategies in mitigating genetic susceptibility to metabolic diseases. In the context of gene–diet interactions, particularly in a South Asian population, this study provides evidence to support the development of personalized nutrition strategies and public health interventions tailored to genetic susceptibility and traditional dietary patterns. Future longitudinal and interventional studies with larger cohorts are essential to validate and expand upon these findings.

## Figures and Tables

**Table 1:** Anthropometric, biochemical, and dietary characteristics of centrally obese and non-centrally obese participants.

Parameter	Mean ± SE		*p* Value
	Non-Centrally Obese (*n* = 54)	Centrally Obese (*n* = 51)	
Age (years)	36.87 ± 0.93	39.25 ± 0.97	0.078
BMI (kg/m^2^)	22.43 ± 0.42	26.81 ± 2.95	**<0.001**
Waist Circumference (cm)	86.25 ± 1.99	80.34 ± 2.95	**0.039**
Hip Circumference (cm)	93.50 ± 2.04	87.85 ± 2.85	0.054
Waist Hip Ratio	0.92 ± 0.01	0.91 ± 0.18	0.297
Fat Mass (kg/m^2^)	23.84 ± 0.86	30.51 ± 0.97	**<0.001**
Systolic Blood Pressure (mm Hg)	116.96 ± 1.99	123.09 ± 2.20	0.019
Diastolic Blood Pressure (mm Hg)	74.74 ± 2.61	74.92 ± 1.80	0.367
Cholesterol (mg/dL)	202.79 ± 4.89	208 ± 4.69	0.208
High-Density Lipoprotein (mg/dL)	42.81 ± 1.19	42.11 ± 1.09	0.360
Low-Density Lipoprotein (mg/dL)	132.24 ± 4.23	135.45 ± 3.89	0.289
Very-Low-Density Lipoprotein (mg/dL)	28.57 ± 2.71	30.78 ± 2.37	0.163
Glucose (mg/dL)	83.40 ± 0.91	89.92 ± 4.33	0.051
Insulin (pmol/L)	54.86 ± 5.55	81.88 ± 7.72	**<0.001**
HbA1c (%)	5.33 ± 0.06	5.52 ± 0.13	0.090
Total Energy (kcal)	2048.93 ± 60.54	2130.23 ± 66.23	0.218
Protein (%)	58.78 ± 2.61	59.44 ± 2.42	0.419
Carbohydrate (%)	355.74 ± 10.16	365.02 ± 11.36	0.319
Fat (%)	50.06 ± 2.85	52.93 ± 2.70	0.378
Fibre (g/day)	16.36 ± 1.07	16.88 ± 1.86	0.932
PUFA (g/day)	3.35 ± 0.23	3.40 ± 0.26	0.717
Genetic Risk Score	0.48 ± 0.06	0.56 ± 0.07	0.616

Data presented as mean ± standard deviation. Independent sample *t*-test was performed to compare the variables between centrally obese and non-centrally obese participants. The *p* value < 0.05 using linear regression analysis (highlighted in bold), shows significant association between the two groups. Centrally obese participants refer to men with WC ≥ 94 cm and women with WC ≥ 80 cm (WHO). Log transformed variables—BMI (Body Mass Index), waist circumference (cm), hip circumference (cm), systolic blood pressure (mmHg), diastolic blood pressure (mmHg), high-density lipoprotein (mg/dL), low-density lipoprotein (mg/dL), very-low-density lipoprotein (mg/dL), glucose (mg/dL), insulin (pmol/L), HbA1c(%), total energy (kcal), protein (%), carbohydrate (%), fat (%), fibre (g/day), PUFA (g/day).

**Table 2:** Association of Genetic Risk Score (GRS) with anthropometric, biochemical, and dietary parameters.

Variables	GRS Groups		*p* Value
	Low Risk (*n* = 50)	High Risk (*n* = 55)	
	Mean ± SE		
BMI (kg/m^2^)	25.49 ± 0.65	23.71 ± 0.48	0.581
Waist Circumference (cm)	80.95 ± 2.80	85.60 ± 2.22	0.105
Hip Circumference (cm)	89.01 ± 2.71	92.34 ± 2.27	0.191
Waist Hip Ratio	0.90 ± 0.14	0.93 ± 0.01	0.528
Fat Mass (kg/m^2^)	28.68 ± 1.17	25.63 ± 0.84	0.371
Systolic Blood Pressure (mm Hg)	122.18 ± 2.38	117.90 ± 1.86	0.874
Diastolic Blood Pressure (mm Hg)	75.84 ± 1.52	73.90 ± 2.72	0.500
Cholesterol (mg/dL)	206.34 ± 5.18	204.72 ± 4.48	0.936
High-Density Lipoprotein (mg/dL)	42.46 ± 1.17	42.49 ± 1.12	0.213
Low-Density Lipoprotein (mg/dL)	135.51 ± 4.63	132.24 ± 3.52	0.954
Very-Low-Density Lipoprotein (mg/dL)	28.36 ± 2.35	30.80 ± 2.71	0.218
Glucose (mg/dL)	90.34 ± 4.41	83.14 ± 0.87	0.577
Insulin (pmol/L)	76.16 ± 7.54	60.55 ± 6.18	0.135
HbA1c (%)	5.567 ± 0.13	5.29 ± 0.05	0.267
Total Energy (kcal)	2058.13 ± 66.64	2115.96 ± 60.52	0.888
Protein (%)	59.79 ± 2.36	58.48 ± 2.65	0.407
Carbohydrate (%)	355.29 ± 10.95	364.76 ± 10.56	0.376
Fat (%)	50.21 ± 2.58	52.58 ± 2.93	0.839
Fibre (g/day)	16.16 ± 1.13	17.02 ± 1.12	0.530
PUFA (g/day)	3.34 ± 0.23	3.41 ± 0.25	0.069

Data presented as mean ± standard error. Linear regression analysis was used to compare the variables between low and high genetic risk groups. *p* < 0.05, statistically significant association between GRS and variables adjusted for confounders. Log transformed variables—BMI (Body Mass Index), waist circumference (cm), hip circumference (cm), systolic blood pressure (mmHg), diastolic blood pressure (mmHg), high-density lipoprotein (mg/dL), low-density lipoprotein (mg/dL), very-low-density lipoprotein (mg/dL), glucose (mg/dL), insulin (pmol/L), HbA1c (%), total energy (kcal), protein (%), carbohydrate (%), fat (%), fibre (g/day), PUFA (g/day).

**Table 3:** Gene–diet interactions between 10-SNP metabolic GRS and dietary factors on central obesity status, and anthropometric and biochemical parameters.

Parameters	Total Energy (kcal)	Protein (%)	Carbohydrate (%)	Fat (%)	Fibre (g)	PUFA (g)
BMI (kg/m^2^)	0.615	0.693	0.572	0.586	0.687	0.478
Waist Circumference (cm)	0.016	**0.025**	**0.004**	0.158	**0.011**	**<0.001**
Waist Hip Ratio	0.558	0.253	0.545	0.262	0.255	0.305
Fat Mass (kg/m^2^)	0.637	0.268	0.424	0.596	0.790	0.907
Systolic Blood Pressure (mm Hg)	0.320	0.373	0.079	0.719	0.241	0.161
Diastolic Blood Pressure (mm Hg)	0.894	0.516	0.880	0.904	0.779	0.970
Cholesterol (mg/dL)	0.337	0.669	0.289	0.350	0.243	0.957
High-Density Lipoprotein (mg/dL)	0.178	0.265	0.464	0.097	0.358	0.606
Low-Density Lipoprotein (mg/dL)	0.231	0.416	0.303	0.088	0.082	0.618
Very-Low-Density Lipoprotein (mg/dL)	0.489	0.863	0.406	0.892	0.894	0.665
Glucose (mg/dL)	0.956	0.872	0.861	0.973	0.996	0.077
Insulin (pmol/L)	0.324	0.069	0.302	0.127	0.190	0.289
HbA1c (%)	0.999	0.997	0.999	0.989	0.999	0.415
Central Obesity	0.222	0.452	0.273	0.215	0.501	0.339

Data is presented as *p*-value for the interactions between GRS and dietary factors on anthropometric and biochemical parameters. The *p* value < 0.05 (highlighted in bold), shows significant gene-diet interactions. Gene–diet interaction models were adjusted for relevant confounders including age, sex, BMI (if not the dependent variable), alcohol use, smoking status, and total energy intake where applicable. Log transformed variables—BMI (Body Mass Index), waist circumference (cm), hip circumference (cm), systolic blood pressure (mmHg), diastolic blood pressure (mmHg), high-density lipoprotein (mg/dL), low-density lipoprotein (mg/dL), very-low-density lipoprotein (mg/dL), glucose (mg/dL), insulin (pmol/L), HbA1c (%), total energy (kcal), protein (%), carbohydrate (%), fat (%), fibre (g/day), PUFA (g/day).

## Data Availability

The original contributions presented in this study are included in the manuscript. Further inquiries can be directed to the corresponding author.

## Supplementary Materials

The following supporting information can be downloaded at: https://www.mdpi.com/article/10.3390/nu17172866/s1, Table S1: Genotype. Major allele and minor allele frequencies and Hardy–Weinberg Equilibrium *p* value of the SNPs chosen for the study. Table S2: Odds Ratios for SNPs used for GRS construction (Outcome: Central Obesity).

## Abbreviations

The following abbreviations are used in this manuscript:

BMI	Body Mass Index
WC	Waist Circumference
GRS	Genetic Risk Score
PUFA	Polyunsaturated Fatty Acids
SNP	Single Nucleotide Polymorphism
FFQ	Food Frequency Questionnaire
SPSS	Statistical Package for the Social Sciences
HWE	Hardy–Weinberg Equilibrium
HDL	High-Density Lipoprotein
LDL	Low-Density Lipoprotein
VLDL	Very-Low-Density Lipoprotein
*PPAR*	Peroxisome Proliferator-Activated Receptor
*SREBP*	Sterol Regulatory Element-Binding Protein
*KASP*	Kompetitive Allele-Specific PCR
GOOD	Genetics of Obesity and Diabetes
*CAPN10*	Calpain 10
*KCNJ11*	Potassium Inwardly-Rectifying Channel Subfamily J Member 11
*TCF7L2*	Transcription Factor 7 Like 2
*FTO*	Fat Mass and Obesity Associated
*MC4R*	Melanocortin-4 Receptor
