# Supplementary material for: High Polyunsaturated Fatty Acid Intake Attenuates the Genetic Risk of Higher Waist Circumference in a Sri Lankan Adult Population
Source: Nutrients. 2025 Sep 4;17(17):2866. doi: 10.3390/nu17172866 (PMC12430655; doi:10.3390/nu17172866)
Supplement: Supplementary file 1 [file nutrients-17-02866-s001.zip › nutrients-3774527-supplementary.pdf]

Supplementary Materials:

Table S1. Genotype. Major allele and minor allele frequencies and Hardy-Weinberg Equilibrium P value of the SNPs chosen for the study.

	Gene Name	rs number	Major/Minor Allele	Major/Minor Allele Frequency (%)	Genotype Frequency (n)	HWE	n
CAPN10	Calpain 10	rs3792267	G/A	82.55/17.45	75/24/6	0.05	105
CAPN10	Calpain 10	rs2975760	T/C	77.83/22.17	63/37/5	0.89	105
CAPN10	Calpain 10	rs5030952	C/T	96.69/3.31	99/6/0	0.76	105
KCNJ11	Potassium inwardly-rectifying channel, subfamily J, member 11 protein	rs5219	C/T	66.50/33.50	49/42/14	0.31	105
TCF7L2	Transcription factor 7 like 2	rs12255372	G/T	73.11/26.89	56/42/7	0.76	105
TCF7L2	Transcription factor 7 like 2	rs7903146	C/T	65.57/34.43	43/52/10	0.31	105
FTO	Fat mass and obesity associated	rs9939609	T/A	21.23/78.77	46/45/14	0.57	105
MC4R	Melanocortin receptor	rs17782313	T/C	67.45/32.55	47/48/10	0.65	105
FTO	Fat mass and obesity associated	rs8050136	C/A	65.57/34.43	46/45/14	0.57	105
MC4R	Melanocortin-4 receptor	rs2229616	G/A	96.23/3.77	97/8/0	0.68	105

CAPN10: Calpain 10; KCNJ11: Potassium Inwardly-Rectifying Channel Subfamily J Member 11; TCF7L2: Transcription Factor 7 Like 2; FTO: Fat Mass and Obesity Associated; MC4R: Melanocortin-4 Receptor.

Table S2. Odds Ratios for SNPs used for GRS construction (Outcome: Central Obesity)

SNP ID	Comparison Group (Dominant Model)	OR (Exp(B))	95% CI (Lower – Upper)	p-value
**rs3792267**	2 risk alleles vs. none	4.32	0.491 – 37.991	0.187
**rs2975760**	2 risk alleles vs. none	0.403	0.045 – 3.634	0.418
**rs5030952**	1 risk allele vs. none	8.368	0.552 – 126.929	0.126
**rs5219**	2 risk alleles vs. none	0.328	0.082 – 1.320	0.117
**rs12255372**	2 risk alleles vs. none	0.679	0.021 – 21.766	0.827
**rs7903146**	2 risk alleles vs. none	4.799	0.259 – 88.879	0.292
**rs9939609**	1 risk allele vs. none	0.760	0.183 – 3.150	0.144
**rs17782313**	2 risk alleles vs. none	0.441	0.086 – 2.259	0.326
**rs8050136**	1 risk allele vs. none	1.161	0.288 – 4.675	0.834
**rs2229616**	1 risk allele vs. none	1.020	0.194 – 5.367	0.981

Central obesity was defined as WC ≥ 94 cm for men and ≥ 80 cm for women. OR = Odds Ratio; CI = Confidence Interval; $p < 0.05$ considered statistically significant. Reference group = Individuals with 0 risk alleles (wild-type homozygotes). Adjusted for age, sex, energy intake, smoking, and alcohol use where applicable.
